# Publisher Correction: The neuroscience of advanced scientific concepts

**DOI:** 10.1038/s41539-021-00114-7

**Published:** 2021-12-08

**Authors:** Robert A. Mason, Reinhard A. Schumacher, Marcel Adam Just

**Affiliations:** 1 Center for Cognitive Brain Imaging, Carnegie Mellon University, Pittsburgh, PA 15213 USA; 2 Department of Psychology, Carnegie Mellon University, Pittsburgh, PA 15213 USA; 3 Department of Physics, Carnegie Mellon University, Pittsburgh, PA 15213 USA

**Keywords:** Language, Education

Correction to: *npj Science of Learning* 10.1038/s41539-021-00107-6, published online 11 October 2021

In this article the affiliation details for Reinhard Schumacher were incorrectly given as ‘Department of Psychology, Carnegie Mellon University, Pittsburgh, PA 15213, USA’ but should have been ‘Department of Physics, Carnegie Mellon University, Pittsburgh, PA 15213, USA’. The original article has been corrected.

