# Supplementary material for: The neuroscience of advanced scientific concepts
Source: NPJ Sci Learn. 2021 Oct 11;6:29. doi: 10.1038/s41539-021-00107-6 (PMC8505455; doi:10.1038/s41539-021-00107-6)
Supplement: Supplementary file 1 — Reporting Summary [file 41539_2021_107_MOESM1_ESM.pdf]

nature portfolio

Corresponding author(s): Robert A. Mason

Last updated by author(s): Sep 2, 2021

Reporting Summary

Nature Portfolio wishes to improve the reproducibility of the work that we publish. This form provides structure for consistency and transparency in reporting. For further information on Nature Portfolio policies, see our Editorial Policies and the Editorial Policy Checklist.

Statistics

For all statistical analyses, confirm that the following items are present in the figure legend, table legend, main text, or Methods section.

n/a	Confirmed	
☐	☒	The exact sample size (*n*) for each experimental group/condition, given as a discrete number and unit of measurement
☐	☒	A statement on whether measurements were taken from distinct samples or whether the same sample was measured repeatedly
☐	☒	The statistical test(s) used AND whether they are one- or two-sided<br>*Only common tests should be described solely by name; describe more complex techniques in the Methods section.*
☐	☒	A description of all covariates tested
☐	☒	A description of any assumptions or corrections, such as tests of normality and adjustment for multiple comparisons
☐	☒	A full description of the statistical parameters including central tendency (e.g. means) or other basic estimates (e.g. regression coefficient) AND variation (e.g. standard deviation) or associated estimates of uncertainty (e.g. confidence intervals)
☐	☒	For null hypothesis testing, the test statistic (e.g. *F*, *t*, *r*) with confidence intervals, effect sizes, degrees of freedom and *P* value noted<br>*Give P values as exact values whenever suitable.*
☐	☒	For Bayesian analysis, information on the choice of priors and Markov chain Monte Carlo settings
☐	☒	For hierarchical and complex designs, identification of the appropriate level for tests and full reporting of outcomes
☐	☒	Estimates of effect sizes (e.g. Cohen's *d*, Pearson's *r*), indicating how they were calculated

Our web collection on statistics for biologists contains articles on many of the points above.

Software and code

Policy information about availability of computer code

Data collection	All data was collected using standard CMRR multi-band pulse sequences.
Data analysis	Matlab version R2014a<br>SPM8<br>Custom Matlab code (most of this code was used in the previously published studies). Additional analysis algorithms are described in the Methods section or will be provided upon request.

For manuscripts utilizing custom algorithms or software that are central to the research but not yet described in published literature, software must be made available to editors and reviewers. We strongly encourage code deposition in a community repository (e.g. GitHub). See the Nature Portfolio guidelines for submitting code & software for further information.

Data

Policy information about availability of data

All manuscripts must include a data availability statement. This statement should provide the following information, where applicable:

- Accession codes, unique identifiers, or web links for publicly available datasets
- A description of any restrictions on data availability
- For clinical datasets or third party data, please ensure that the statement adheres to our policy

All data Will be made available from a repository upon acceptance.

Field-specific reporting

Please select the one below that is the best fit for your research. If you are not sure, read the appropriate sections before making your selection.

☒ Life sciences ☐ Behavioural & social sciences ☐ Ecological, evolutionary & environmental sciences

For a reference copy of the document with all sections, see nature.com/documents/nr-reporting-summary-flat.pdf

Life sciences study design

All studies must disclose on these points even when the disclosure is negative.

Sample size	The sample size for the main analysis (10 faculty members) matches the size used in our previous study (Mason & Just 2016) and many similar studies cited in the manuscript. Classification accuracies were reliable above chance in all participants in both with and between-participant analyses.
Data exclusions	Data from three additional participants were not included due to reasons withheld here for privacy in this small group.
Replication	Classification analyses are reported for varying subsets of participants and concepts. Concepts in the three withheld participants were reliably identified in the within participant design and one was reliably classified in the between participant design before the reason for being excluded became known.
Randomization	This is not relevant as all participants were members of the physics faculty.
Blinding	This is not relevant as all participants were members of the physics faculty. For the faculty-student contrast the machine learning classifier was blinded to the test-participant.

Reporting for specific materials, systems and methods

We require information from authors about some types of materials, experimental systems and methods used in many studies. Here, indicate whether each material, system or method listed is relevant to your study. If you are not sure if a list item applies to your research, read the appropriate section before selecting a response.

Materials & experimental systems

n/a	Involved in the study	
☒	☐	Antibodies
☒	☐	Eukaryotic cell lines
☒	☐	Palaeontology and archaeology
☒	☐	Animals and other organisms
☐	☒	Human research participants
☒	☐	Clinical data
☒	☐	Dual use research of concern

Methods

n/a	Involved in the study	
☒	☐	ChIP-seq
☒	☐	Flow cytometry
☐	☒	MRI-based neuroimaging

Human research participants

Policy information about studies involving human research participants

Population characteristics	Ten right-handed faculty members (all male, between the ages of 35 and 67) from the Carnegie Mellon University Physics Department participated in the fMRI scanning task. All scanned participants gave signed informed consent approved by the Carnegie Mellon Institutional Review Board. The native languages of the participants included English, German, and Mandarin Chinese. Data from three additional participants were not included due to reasons withheld here for privacy in this small group. Six additional faculty members (5 male, 1 female, between the ages of 40 and 79) from the Physics Department who had not been scanned participated as physics experts to rate the concepts according to the postulated dimensions.
Recruitment	Carnegie Mellon University physics faculty participants were contacted by the authors and assessed for interest and MRI compatibility.
Ethics oversight	Carnegie Mellon Institutional Review Board

Note that full information on the approval of the study protocol must also be provided in the manuscript.

Magnetic resonance imaging

Experimental design

Design type

The participants were instructed to actively and re-iteratively think about the properties of the presented concept. To promote their consideration of a consistent set of properties or features across the six presentations of each concept, participants were asked to write down two or three properties of their choosing for each item prior to the scanning session (for example, the properties for the term velocity might be vector quantity, movement related, and directional).

Design specifications

The stimuli were 45 physics terms with five concepts from each of nine physics topic areas: (particle/nuclear physics, astrophysics, condensed matter, special relativity, classical mechanics, quantum mechanics, elementary classical mechanics, elementary energy/electricity, elementary light/sound). These concepts were selected to be representative of the knowledge of any Ph.D.-level academic physicist, irrespective of research specialization. The 15 concepts from three elementary categories were a subset of those included in a previous investigation of physics concepts in college students (11).

The set of 45 concepts was presented six times (in six different random permutation orders of the 45 items). Each concept label was visually presented on a video screen for 4 sec during which the participant thought about the properties of the concept, followed by a 6 sec rest period, during which the participant fixated on a shrinking and gradually disappearing blue ellipse displayed in the center of the screen. There were seven additional longer presentations (17 sec) of a shrinking ellipse distributed across the session to provide a baseline measure of brain activity.

Behavioral performance measures

This task is naturalistic, but it is fairly demanding and could invite inattention. A sufficiently high level of classification accuracy is used to ensure that all participants paid attention and performed the task throughout the experiment.

Acquisition

Imaging type(s)

functional

Field strength

3

Sequence & imaging parameters

Functional images were acquired on a Siemens Verio (Erlangen, Germany) 3.0T scanner at the Scientific Imaging and Brain Research Center of Carnegie Mellon University using a gradient echo planar imaging (EPI) pulse sequence with TR = 1000 ms, TE = 25 ms and a 60° flip angle. Twenty 5-mm thick AC-PC aligned slices were imaged with a gap of 1 mm between slices using a 32-channel head coil. The acquisition matrix was 64 × 64 with 3.125-mm × 3.125-mm × 5.0-mm in-plane resolution. Images were corrected for slice acquisition timing, motion, and linear trend, and were normalized to the Montreal Neurological Institute (MNI) template without changing voxel size (3.125 × 3.125 × 6 mm) using SPM8 (Wellcome Dept. of Cog. Neurology).

Area of acquisition

Twenty 5-mm thick AC-PC aligned slices were imaged with a gap of 1 mm between slices using a 32-channel head coil.

Diffusion MRI ☐ Used ☒ Not used

Preprocessing

Preprocessing software

Images were corrected for slice acquisition timing, motion, and linear trend using SPM8 (Wellcome Dept. of Cog. Neurology).

Normalization

Images were were normalized to the Montreal Neurological Institute (MNI) template without changing voxel size (3.125 × 3.125 × 6 mm) using SPM8 (Wellcome Dept. of Cog. Neurology).

Normalization template

Montreal Neurological Institute (MNI) template

Noise and artifact removal

none

Volume censoring

None

Statistical modeling & inference

Model type and settings

the postulated relation of each concept to each of the four dimensions was estimated by the mean ratings described above. A linear regression model with four predictor variables developed a mapping between the ratings along the four dimensions (factors) of all but one concept and the mean fMRI activation level in each of the 30 factor clusters/locations for that concept.

Effect(s) tested

The model weights were then applied to the ratings of the left-out concept to predict the activation pattern for that concept.

Specify type of analysis: ☐ Whole brain ☒ ROI-based ☐ Both

Anatomical location(s)

Locations were defined on the basis of a factor analysis of the three most accurately classified participants. These participants were not included in the test set.

Statistic type for inference
(See Eklund et al. 2016)

The predictive model was evaluated in two ways: (1) the similarity of the model predictions to the observed activation patterns, which was assessed using R2 (the goodness of fit as the proportion of the variation in the observed activation data explained by the predictions of the model); and (2) the ability to distinguish among concepts, which was assessed using classification accuracy based on the distance between the predicted and observed activation of each concept.

Correction

The mean normalized rank accuracy for classification used permutation testing.

Models & analysis

n/a	Involved in the study
☒	☐ Functional and/or effective connectivity
☒	☐ Graph analysis
☐	☒ Multivariate modeling or predictive analysis

Multivariate modeling and predictive analysis

Gaussian Naïve Bayes (GNB) classifiers were used to identify the 45 physics concepts (for an overview of the GNB classifier cross-validation as applied to fMRI data see 41 and the Supplemental Methods). The classifiers were trained using the activation levels of stable voxels from only a subset of the data (the training set), and then tested on the remaining independent data (the test set) using a cross-validation procedure. For the within-participant classification, the training set on each fold consisted of the data for each item (i.e. the activation levels of the selected voxels) from four of the six presentations and the test set consisted of the mean of the data from the remaining two presentations. For cross-participant classification, the classifier was trained on the data from nine participants and tested on the 10th, left-out participant. In the latter analysis, each participant's data was averaged over the six presentations. Then the 120 voxels with the most similar activation profiles across the 45 concepts (assessed with correlation) across the nine participants in the training set were selected as features for the classifier.
