# Supplementary material for: The neuroscience of advanced scientific concepts
Source: NPJ Sci Learn. 2021 Oct 11;6:29. doi: 10.1038/s41539-021-00107-6 (PMC8505455; doi:10.1038/s41539-021-00107-6)
Supplement: Supplementary file 2 — Supplementary Information [file 41539_2021_107_MOESM2_ESM.pdf]

The neuroscience of advanced scientific concepts

Authors: Robert A. Mason [I*], Reinhard A. Schumacher [II], Marcel Adam Just [I]

Supplementary Materials

Supplementary Table 1. List of voxel clusters, their centroids (MNI coordinates) and size (in voxels) associated with each factor (factor labels appear in bold).

**Measurable Magnitude**		*x*	*y*	*z*	cluster size
Left	Middle Temporal	-51	-59	6	58
Left	Inferior Parietal	-32	-47	48	32
Left	Supramarginal	-60	-42	35	20
Left	Superior Frontal	-25	-8	57	15
Left	Superior Frontal	-14	58	23	11
Left	Precuneus	-11	-69	55	11
Left	Occipital	-33	-85	33	10
Left	Superior Parietal	-25	-70	52	9
**Mathematical formulation**					
Left	Superior Parietal	-22	-72	45	7
Left	Supplementary Motor	0	9	63	5
Right	Precentral	40	4	50	12
Right	Inferior Frontal (pars triangularis)	52	34	27	5
**Periodicity**					
Left	Inferior Temporal	-52	-60	-14	5
Left	Precuneus	-1	-72	40	5
Right	Inferior Frontal (pars opercularis)	48	10	26	22

Right	Middle Temporal	54	-54	14	8
Right	Precuneus	15	-70	45	5
Right	Angular	40	-60	48	5
**Classical vs. post-Classical**					
Left	Precuneus	0	-63	46	31
Left	Inferior Parietal	-36	-65	52	18
Left	Inferior Frontal (Rolandic opercularis)	-50	4	13	11
Left	Supramarginal	-56	-51	23	11
Left	Supplementary Motor	-12	16	62	10
Left	Postcentral	-58	-8	41	5
Left	Medial Frontal	-12	54	26	4
Left	Medial Frontal	-15	53	10	2
Right	Inferior Temporal	59	-59	-11	15
Right	Superior Parietal	26	-65	52	15
Right	Middle Temporal	52	-32	-7	8
Right	Inferior Parietal	40	-53	52	7
Right	Middle Frontal	48	43	22	7
Right	Inferior Frontal (pars opercularis)	50	17	38	5
Right	Middle Frontal	41	8	58	3
Right	Fusiform	28	-35	-17	2
Right	Inferior Orbital Frontal	45	28	-35	2

Supplementary Methods

Selecting Voxels with Stable Activation Patterns. The analyses generally focus on a small subset of all the voxels in the brain. A voxel's stability was computed as the average pairwise correlation between its 45-concept activation profiles across the multiple presentations that serve as input for a given model. The machine learning classification is based upon a set of stable voxels (100 per lobe for factor analysis; 120 over the entire brain or the brain excluding the occipital cortex for both the within and between participant classification analyses). In the majority of the analyses, the focus is on small volumes in which many of the participants have stable voxels. These small volumes are determined through a 2-stage factor analysis.

Factor Analysis of the fMRI data. To reduce the dimensionality of the neural activity associated with the stimulus items to a modest number of components, a multi-level exploratory factor analysis (FA) procedure will be applied (this technique is graphically presented as Figure 1 in: Just MA, Cherkassky VL, Buchweitz A, Keller TA, Mitchell TM (2014) Identifying Autism from Neural Representations of Social Interactions: Neurocognitive Markers of Autism. PLoS ONE 9(12): e113879. doi:10.1371/journal.pone.0113879). A Matlab implementation of a principal factor analysis algorithm (equivalent to the SAS v. 9.2 FA procedure) including varimax rotation will be used. Each neural factor is expected to consist of a common activation pattern distributed over approximately 4-6 brain locations. Each individual's neural representation will be reduced to a small set of voxels in sets of clusters that respond similarly. A second level FA will reduce the set of factors to those most common across individuals. The result is a small set of cognitively meaningful group-level factors. For this data set, when a cluster was unassigned to a factor in the second level, or when the second level factor was uninterpretable, those voxels were submitted to a second two level factor analyses.

Machine learning (ML) classification. For the ML classification or multi voxel pattern analysis (MVPA), the PSC of the stable voxels was computed. Gaussian Naive Bayes (GNB) classifiers whose features are the activation levels of stable voxels in small regions defined by their high loading on a factor in the factor analysis of the activation data will be used to identify the concepts. The classifiers will be trained using voxels from only a subset of the data (the training set), and then tested on the remaining data (the test set) using a cross-validation procedure to ensure unbiased estimation of the classification error in both a within- and between-participant design.

Classifiers will be trained to identify activation states associated with thinking about the concepts using the evoked patterns of functional activity. Classifiers are functions f of the form: f: mean_PSC → Yj, j={1, …,m}, where Yj is one the concepts, and where mean_PSC is a vector of mean PSC voxel activations associated with a set of factor-determined spherical volumes .

The Gaussian Naïve Bayes (GNB) pooled variance classifier is a generative classifier that models the joint distribution of a class Y (e.g. concepts) and attributes (spheres of voxels), and assumes the attributes $X_1,\ldots,X_n$ are conditionally independent given Y. The classification rule is:

$$Y \leftarrow \arg\max_{y_j} P(Y = y_j) \prod_{i}^{n} P(X_i \mid Y = y_j),\ j = 1,2,\ldots,m,$$

where m is the number of classes and n is the number of spheres.

The **rank accuracy** (hereafter, simply accuracy) of the classification is the normalized rank of the correct label in the classifier's posterior-probability-ordered list of classes. If the classifier were operating at chance, the correct label would on average appear in the middle of the ranked list, producing a chance level accuracy of .50. Accuracies are calculated for each item in each fold and then averaged across folds, and then across items. Significance levels are obtained using

random permutation testing (for the 45-class classification). In the case of classifying membership in two groups, simple accuracy is used and a binomial distribution is used to assess significance levels.
